# Acute Kidney Injury Urinary Biomarker Time-Courses

**DOI:** 10.1371/journal.pone.0101288

**Published:** 2014-07-09

**Authors:** John W. Pickering, Zoltán H. Endre

**Affiliations:** 1 Department of Medicine, University of Otago Christchurch, Christchurch, New Zealand; 2 Department of Nephrology, Prince of Wales Clinical School, University of New South Wales, Sydney, Australia; University Medical Center Groningen and University of Groningen, Netherlands

## Abstract

Factors which modify the excretion profiles of acute kidney injury biomarkers are difficult to measure. To facilitate biomarker choice and interpretation we modelled key modifying factors: extent of hyperfiltration or reduced glomerular filtration rate, structural damage, and reduced nephron number. The time-courses of pre-formed, induced (upregulated), and filtered biomarker concentrations were modelled in single nephrons, then combined to construct three multiple-nephron models: a healthy kidney with normal nephron number, a non-diabetic hyperfiltering kidney with reduced nephron number but maintained total glomerular filtration rate, and a chronic kidney disease kidney with reduced nephron number and reduced glomerular filtration rate. Time-courses for each model were derived for acute kidney injury scenarios of structural damage and/or reduced nephron number. The model predicted that pre-formed biomarkers would respond quickest to injury with a brief period of elevation, which would be easily missed in clinical scenarios. Induced biomarker time-courses would be influenced by biomarker-specific physiology and the balance between insult severity (which increased single nephron excretion), the number of remaining nephrons (reduced total excretion), and the extent of glomerular filtration rate reduction (increased concentration). Filtered biomarkers have the longest time-course because plasma levels increased following glomerular filtration rate decrease. Peak concentration and profile depended on the extent of damage to the reabsorption mechanism and recovery rate. Rapid recovery may be detected through a rapid reduction in urinary concentration. For all biomarkers, impaired hyperfiltration substantially increased concentration, especially with chronic kidney disease. For clinical validation of these model-derived predictions the clinical biomarker of choice will depend on timing in relation to renal insult and interpretation will require the pre-insult nephron number (renal mass) and detection of hyperfiltration.

## Introduction

Proteomics and genomics have identified many candidate urinary biomarkers of acute kidney injury (AKI). The clinical utility of these biomarkers is dependent on the time at which they are sampled following renal injury [1], [2]. Some biomarkers have very short time-courses [2] with an early peak followed by rapid decline, for example 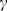-glutymaltranspeptidase (GGT). Others peak later and have a slower decline, for example Neutrophil Gelatinase Associated Lipocalin (NGAL) [2]–[4]. Biomarker analysis has concentrated on the association between biomarker concentration and AKI defined by increased plasma creatinine concentration. Little is known about the comparative influence of change in glomerular filtration rate (GFR) and biomarker generation on urinary biomarker concentrations and time-courses. We mathematically modeled the single nephron excretion time-course for a step decrease in GFR for three classes of urinary biomarker, namely those which are: (i) pre-formed in the tubules and released into the urine during injury, (ii) induced or upregulated within the tubules, and (iii) first filtered by the glomerulus and possibly reabsorbed within the tubules. We then describe how urinary concentrations of these biomarkers depend on the pre-insult state of the kidney and the extent of both nephron loss and the GFR reduction in AKI.

## Methods

The urinary biomarker concentration depends on the total mass of biomarker released into the urine and the total urine flow. The single nephron biomarker concentration varies as the mass of biomarker excreted into the urine (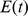) divided by the single nephron urine flow rate (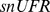).

### Single Nephron urine flow rate

The single nephron urine flow rate (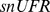) is the single nephron GFR (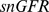) minus the rate of water absorption in the proximal tubule (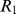) and in the distal tubule and collection duct (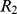):
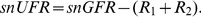
(1)


While 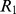 may vary along with change in sodium reabsorption and 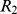 may increase with increased anti-diuretic hormone activity, we have combined the two and modelled total reabsorption (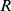) as a percentage of GFR.

### Pre-formed biomarkers

Let 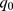 be the mass of pre-formed biomarker prior to an insult to the nephron. Let the duration of insult be 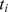 (from time 0). Under steady state conditions the rate of biomarker excretion into the nephron tubule equals the production rate. Let 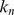 be the normal rate constant for both excretion and production of the preformed biomarker. At equilibrium,
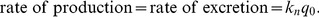
(2)


During an insult (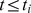), the rate of excretion depends on the available mass of biomarker, 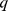, and the rate at which the insult results in further biomarker excretion (rate constant 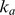). Therefore,
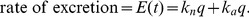
(3)


Therefore, the net rate of change in biomarker mass is,
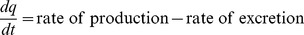
(4)

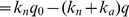
(5)which has the solution
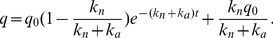
(6)


Substituting for 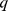 in equation 3 gives,
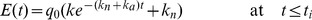
(7)


Following the insult (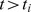),
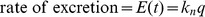
(8)

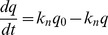
(9)

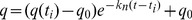
(10)

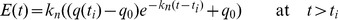
(11)


### Induced biomarkers

**Figure 1:**
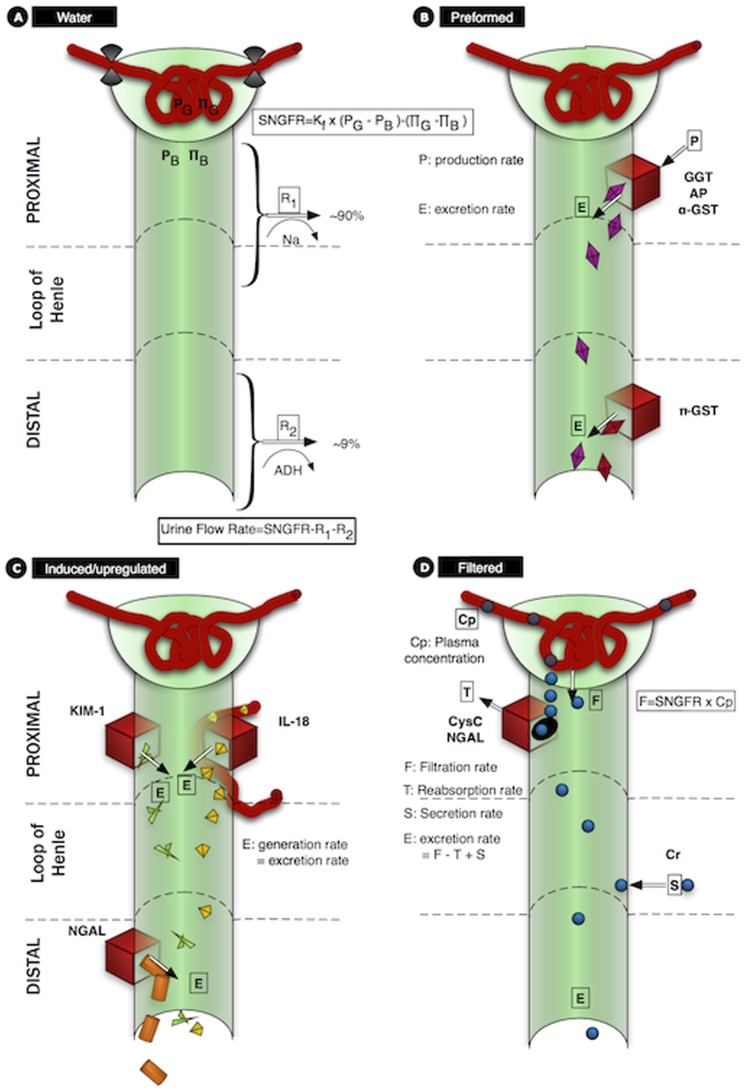
Single Nephron Models. A: Water reabsorption. B: Preformed biomarkers such as 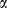-GST are excreted at a rate (E) dependent on the initial (remaining) mass of biomarker and the production rate (P) of new biomarker. C: Induced biomarkers such as KIM-1 are excreted at a rate approximating a log-normal distribution. D: Filtered biomarkers such as Cystatin C are filtered at a rate (F) dependent on the plasma concentration and single nephron GFR and then reabsorbed at a rate dependent on the number of available transporters (T). Some biomarkers are also secreted (S). The final excretion rate is the sum of the gains minus the losses. The final concentration depends on the excretion rate divided by the urine flow rate. Modified after [34].

Induced biomarker gene expression after renal insult follow approximate log-normal distributions [5], [6]. For the purpose of these simulations we assume that protein production follows mRNA expression. Therefore, we modelled the excretion of induced or up-regulated biomarkers, Figure 1c, as log-normal function following the start of insult,
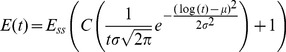
(12)where 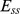 is the steady state excretion rate in the absence of injury, 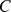 is a constant which scales the total excretion, 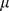 and 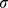 are the mean and standard deviation.

The mean and standard deviations in equation 12 may be determined from the time from insult until peak biomarker excretion 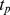 and the time by which half of the total biomarker is excreted, 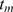,
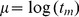
(13)

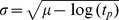
(14)


### Filtered biomarkers

Let 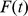 be the rate the biomarker filters through the glomerulus, 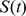 the secretion rate, 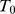 the normal (pre-insult) kidney maximum biomarker reabsorption rate, 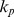 the rate constant for production of receptors (eg megalin or cubulin), and 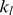 the rate constant for loss of receptors. Normally 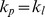 which means that the maximum absorption rate is 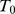. If 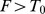 then the excretion rate will be,
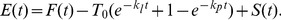
(15)


The rate of filtration may vary according to either a change in systemic rate of production of the biomarker or a change in GFR. The secretion rate may be zero, a constant, or in proportion to the plasma concentration of the biomarker. If the latter then 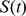 is proportional to 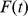 because both are proportional to the plasma concentration.

### Single nephron scenarios

For each biomarker class three scenarios were constructed: (i) the extreme of no change in rate of single nephron biomarker excretion, but loss of 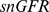 by 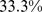, 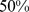, or 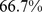 for 48 hours (so called 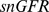 positive/Biomarker negative), (ii) the extreme of no change in 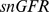 but increases in biomarker excretion (so called 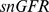 negative/Biomarker positive), (iii) loss of 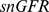 and increase in biomarker excretion. For the pre-formed biomarker we compared the single nephron excretion time-courses for three durations of insults, namely 1 hour, 6 hours and 18 hours. For the induced biomarker we modelled peak biomarker excretion at 6 hours an 12 hours following insult, and allowed for half the total excretion to occur over 12, 15 or 24 hours. For the filtered biomarker we assumed a plasma half-life of 2 hours (e.g. as for Cystatin C) and varied the reabsorption factor through varying 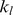. All time-courses are shown as fold increases from pre-insult single nephron urinary excretion.

### Multiple nephron scenarios

Three kidney models were built,

1. **Healthy**: Two million normally functioning nephrons with pre-insult GFR (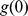) of 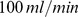. Reabsorption (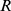) was assumed to be 99% of GFR prior to injury, increasing to 99.5% with reduction of GFR. This effectively maintains urine output for up to a 50% reduction in GFR.
2. **Non-diabetic hyperfilteration**: Normal pre-insult GFR (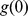) of 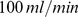 with 1.33 million nephrons. To maintain normal GFR these nephrons have increased filtration (hyperfilteration) by an average of 50%. Reabsorption was defined as for the Healthy kidney model.
3. **CKD**: Reduced pre-insult GFR (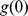) of 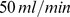 with 0.67 million nephrons. These nephrons have increased filtration (hyperfilteration) by an average of 50%. Reabsorption was assumed to be 99.5% of GFR prior to injury changing to 99.75% with reaction of GFR. This effectively maintains urine output for up to a 50% reduction in GFR.

In the Healthy kidney 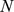 nephrons with 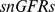 normally distributed around a mean, 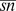. Therefore,
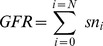
(16)

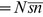
(17)where 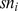 is the 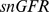 for the 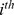 nephron. The 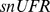 is 1% of the 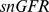. Healthy kidneys are assumed to have the capacity to hyperfilter physiologically, for example following a protein meal or in pregnancy. A similar phenomenon with afferent arteriolar vasodilation occurs in early diabetic kidney disease [7].

**Figure 2:**
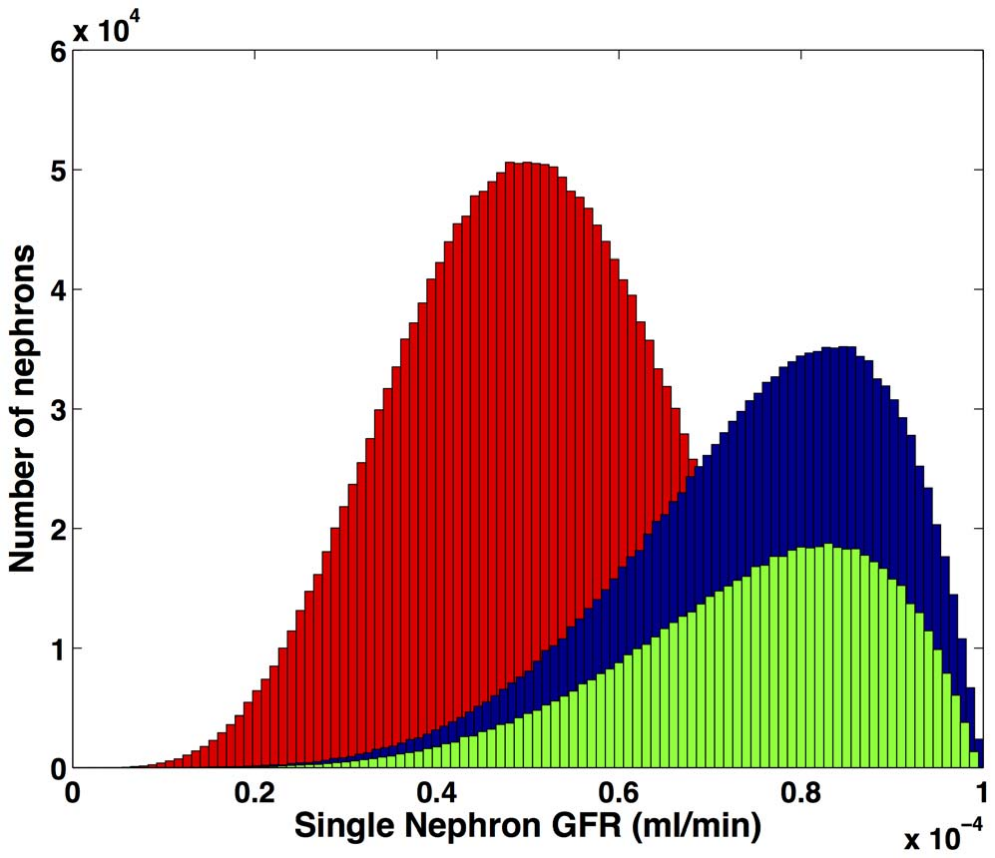
The distribution of single nephron GFRs. Histograms for the Healthy model (dark gray; 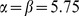), Hyperfilter model (black; 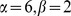) and CKD model (light gray; 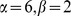). The Healthy model had 2,000,000 nephrons and GFR of 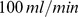 (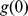), the Hyperfilter model maintained a GFR of 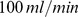 with 33.3% fewer nephrons, and the CKD model had a reduced GFR of 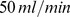 with 66.7% fewer nephrons.

In a non-diabetic hyperfiltering kidney there is assumed loss of function of 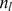 nephrons. The remaining nephrons are hyperfiltering such that the 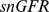 distribution is no longer normal but skewed towards a distribution of greater single nephron GFRs to compensate for the loss of nehprons [8], [9] (see Figure 2). We set the maximum possible snGFR as twice 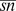 and modelled the 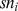 distribution as a beta-function. In the sub-clinical stages of CKD (the Hyperfilter model) we assume that hyperfiltration compensates for nephron loss (up to 33.3% loss of nephrons compensated for by 50% average increase in 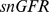, [10]). In the CKD model we assume there has been a further loss of half the remaining nephrons. Although the nephrons are still hyperfiltering, GFR is halved.

The Beta function used in equation 19

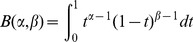
(18)



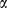
 and 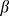 were chosen so that the maximum single nephron GFR was twice the mean and so as to maintain a total GFR of 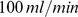 given a loss of one-third of nephrons (hyperfilter model) or 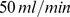 given a loss of two-thirds of nephrons (CKD model).
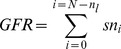
(19)

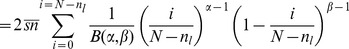
(20)


For each of the three kidney scenarios sixteen biomarker concentration profiles were constructed based on the combination filtration pressure loss (4 scenarios) and nephron loss (4 scenarios),

**1. Filtration pressure loss**: The insult caused uniform loss of filtration pressure for all nephrons, causing a reduction in GFR. GFR was assumed to reduce by 0%, 33.3%, 50%, or 66.7% for 48 hours.

**2. Nephron loss**: The insult caused loss of nephrons with or without hyperfiltration. Nephron loss was assumed to be 0%, 33.3%, 50%, or 66.7%.

The biomarker urinary concentration (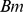) is the sum of each nephron's excretion rate divided by its 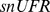:
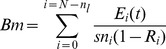
(21)where 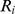 is the proportional reabsorption rate for nephron 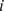 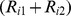. At time zero prior to insult (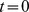) we set 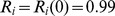 for all nephrons in the Healthy model, thus maintaining a typical UFR of 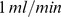. Urine output is commonly maintained in CKD patients. Therefore we set 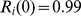 for all the nephrons in the Hyperfilter model and 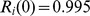 for all the nephrons in the CKD model. We calculated for each of the 16 scenarios the maximum fold increase in biomarker concentration produced contour plots of lines of the same fold increase in biomarker concentration (iso-intensity lines) on a grid of GFR vs nephron number using Matlab function *contourf* to interpolate between points on the grid.

### Urinary NGAL: a case study

Urinary NGAL concentrations derive from both induced biomarker production in the distal tubules and loop of Henle, and from filtered NGAL that has not been reabsorbed in the proximal tubules. For this reason and because NGAL is one of the most promising of structural injury biomarkers, we modelled the time-course as a special case. There are few studies in non-diseased adults from which to determine baseline NGAL concentrations. We chose 38 ng/ml for plasma NGAL, which was the mean concentration of a control group of normal adults [11] and 20 ng/ml for urinary NGAL, which was the median concentration in a study of a healthy population [12]. The urinary concentration depends on the plasma concentration which in turn depends on the change in filtration rate and NGAL production rate. The volume of distribution of NGAL has not been measured, but it is known to be distributed over the plasma and its half-life is short, approximately 15 minutes [13]. We therefore, used a plasma volume of 3000 ml as the volume of distribution. We chose the scenario of a two-thirds reduction in GFR with no further loss of nephron number for this case study.

All calculations were performed in Matlab (Matlab 2012b, MathWorks Inc., Natick, MA, USA).

## Results

### Single nephron scenarios

#### Pre-formed

**Figure 3:**
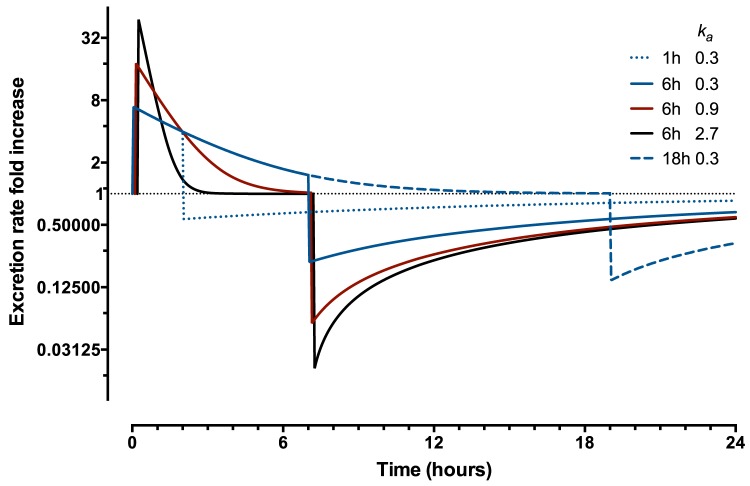
Preformed Biomarkers: single nephron time-course. Fold increase in excretion rate of a pre-formed biomarker for a duration of insult of 1-hour (dotted line), 6-hours (solid lines), 18-hours (dashed line). 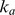 is the rate constant for additional excretion during insult (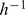). In all cases the excretion rate falls below the pre-AKI excretion rate at the end of the insult because the excretion rate is proportional to the mass of remaining pre-formed biomarker. It is assumed that the biomarker along with the brush border is regenerated at a constant rate, 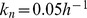.

AKI results in a rapid loss of the mass of pre-formed biomarker (Figure 3). This is manifest by a many fold increase in excretion rate that exponentially declines during the insult as the pre-formed mass is released into the urine. Should the insult cease before the pre-insult mass is excreted, then the excretion rate drops below the pre-insult excretion rate because the mass available for excretion is smaller than pre-insult. As the mass of biomarker slowly recovers (assuming no permanent damage to the tubule) the rate of excretion increases until it reaches the pre-AKI equilibrium rate.

#### Induced

**Figure 4:**
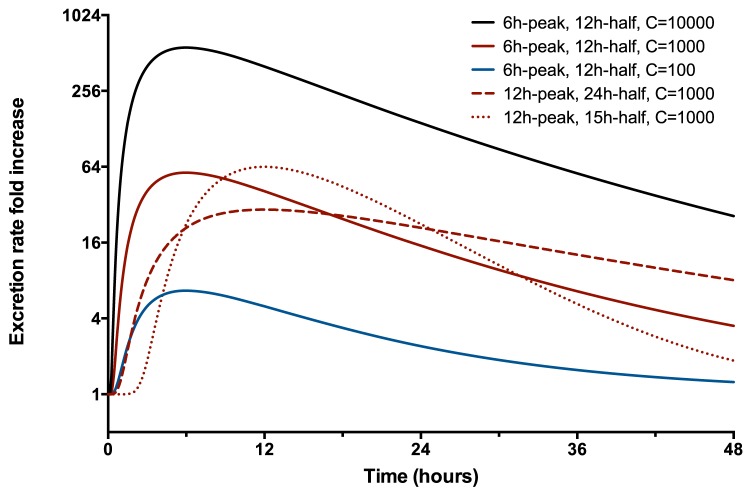
Induced Biomarkers: single nephron time-course. Fold increase in excretion rate of an induced biomarker. Solid lines represent different scaling factors (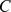) representing greater total damage for a biomarker that peaks at 6 hours and half the total excretion occurs within 12 hours. The dashed line represents the scaling factor of 1000 with a peak at 12 hours and half the total excretion in 24 hours. The dotted line is as for the dashed line but with half the total excretion in 15 hours.

AKI induces some biomarkers to be produced with a portion lost into the tubular filtrate. Pre-AKI excretion rates of induced biomarkers are normally very low and there is a many-fold increase in excretion rate over the space of a few hours (Figure 4). The rate of increase and subsequent decline will vary depending on the biomarker.

#### Filtered

**Figure 5:**
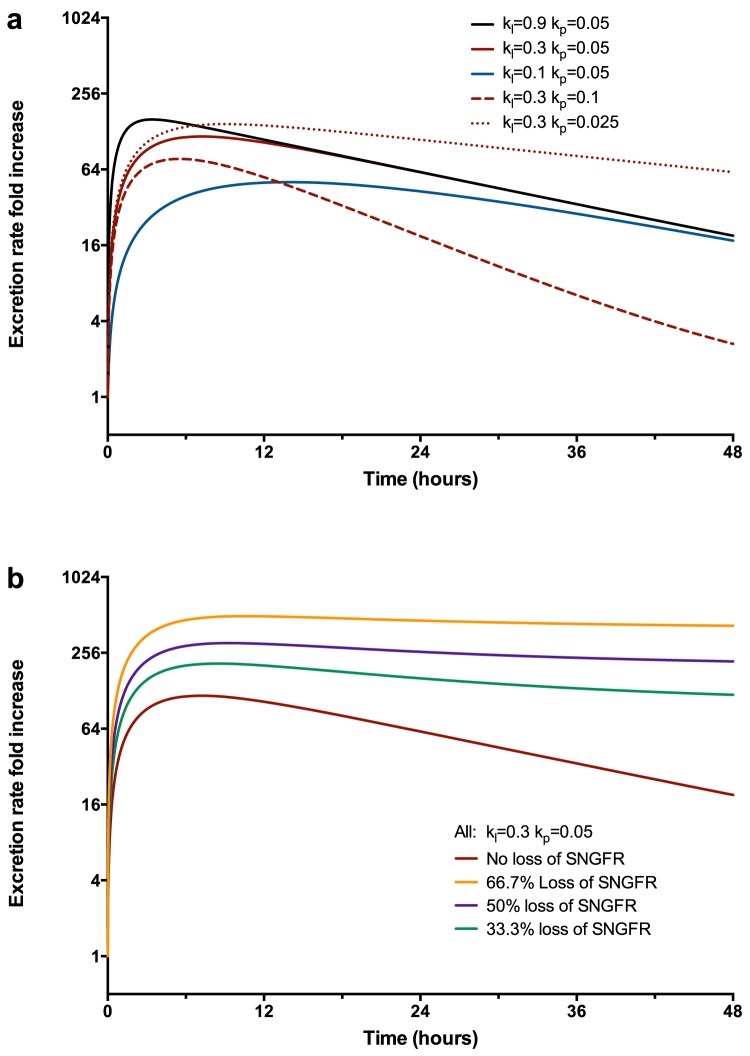
Filtered Biomarkers: single nephron time-course. Fold increase in excretion rate of a filtered biomarker. (a) Solid lines represent different rates of loss of reabsorption receptors. Larger 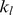 represents greater total damage. Dotted and Dashed lines illustrate the difference the regeneration (production) rate of of the reabsorption receptors makes to the excretion rate. (b) Following a reduction of 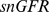 and assuming a plasma half-life of 2 hours (e.g. Cystatin C).

If there is damage to the reabsorption mechanism of a biomarker that is usually (almost) totally reabsorbed within the proximal tubule, then there will be a many fold increase in urinary excretion of that biomarker assuming that the filtration rate has not changed (Figure 5a). Of the three biomarker classes, only the filtered biomarker excretion rate will change because of a change in GFR (Figure 5b) (not to be confused with change induced by injury which reduces GFR). This has the effect of maintaining a greater fold increase over a longer duration.

### Multiple nephron scenarios

For each biomarker class we present contour plots for each kidney type showing the maximum fold increase in biomarkers relative to the pre-insult Healthy kidney model biomarker concentration.

#### Pre-formed

**Figure 6:**
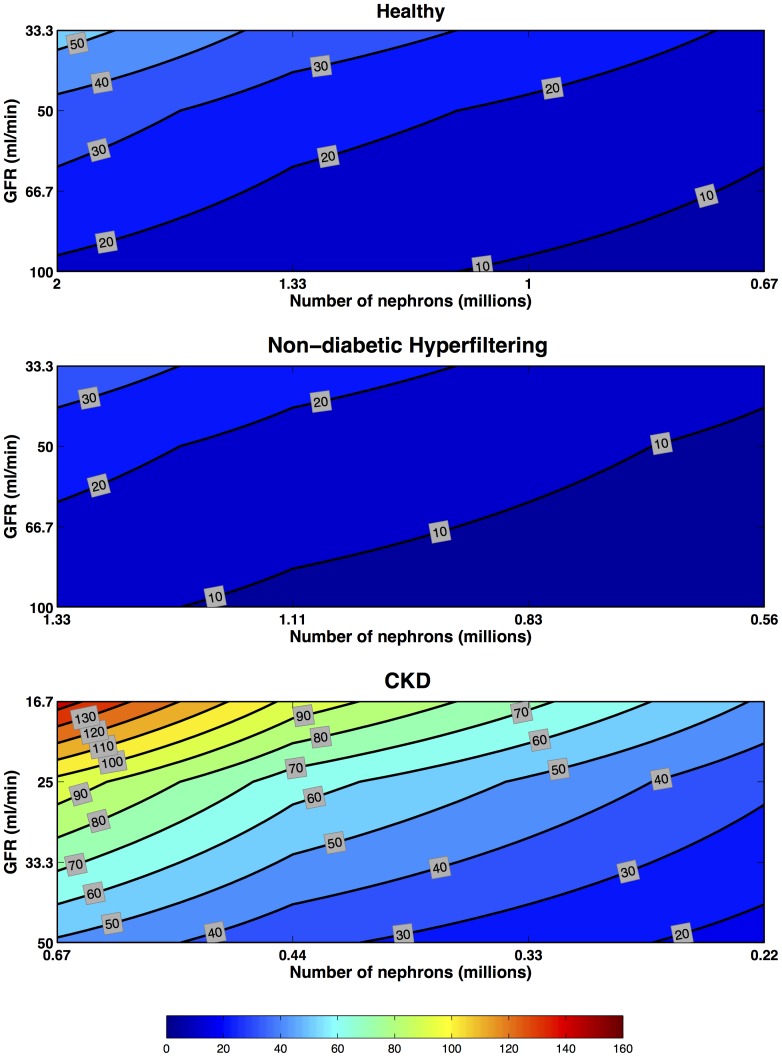
Preformed Biomarkers: multiple-nephron maximum fold concentration increase. Preformed biomarker concentration increases in Healthy, Hyperfiltering and CKD kidneys. Each line of iso-intensity represents the maximal fold increase of a preformed biomarker relative to the pre-injury concentration in a Healthy Kidney with 2 million nephrons and a GFR of 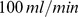. Four GFR scenarios (no change in GFR [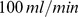 for Healthy and Hyperfiltering kidneys, and 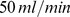 for CKD kidney], one-third, one-half, and two-thirds reduction in GFR) were combined with four nephron number scenarios (no loss [2 M for Healthy, 1.33 M for Hyperfiltering, and 0.67 M for CKD kidneys], one-third, one-half, and two-thirds loss) to produce 16 scenarios. From each of these the maximum fold increase in biomarker concentration was extracted. The iso-intensity lines of fold increase were then interpolated. All scenarios were for a period of AKI of 6 hours, a rate constant of 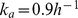 for additional excretion of the biomarker, and a brush border generation rate 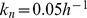. Note, the maximum fold-increase in concentration for each scenario occurs at the same time point, namely immediately following insult.

The maximum concentrations decreased with decreasing number of filtering nephrons and increased with decreasing GFR in each scenario (Figure 6). There is an approximately linear relationship between GFR and nephron number such that a reduction of 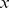
% in GFR and 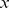
% in nephron number will maintain the same maximal concentrations. The Non-diabetic Hyperfiltering scenario concentrations were lower than in the Healthy scenario at the same GFR's because of fewer nephrons. Conversely concentrations were higher in CKD scenarios despite fewer remaining nephrons because urine output was reduced because of lower GFR.

#### Induced

**Figure 7:**
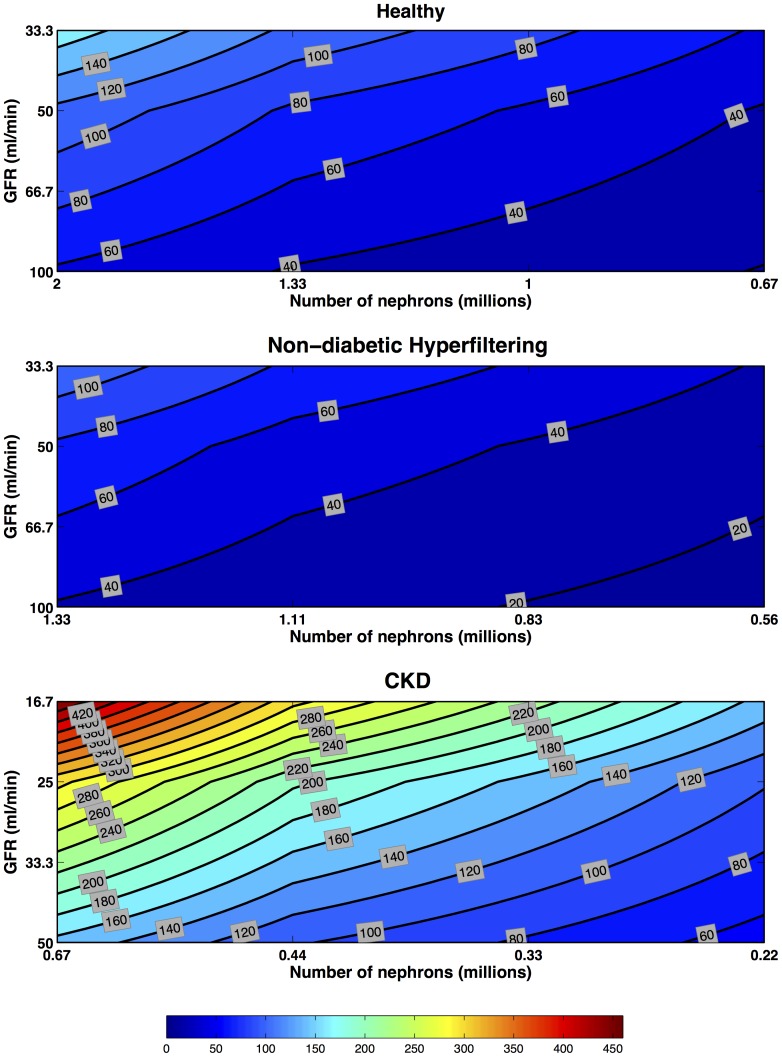
Induced Biomarkers: multiple-nephron maximum fold concentration increase. Induced biomarker concentration increases in Healthy, Hyperfiltering and CKD kidneys. Each line of iso-intensity represents the maximal fold increase of a preformed biomarker relative to the pre-injury concentration in a Healthy Kidney with 2 million nephrons and a GFR of 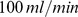. Four GFR scenarios (no change in GFR [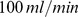 for Healthy and Hyperfiltering kidneys, and 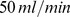 for CKD kidney], one-third, one-half, and two-thirds reduction in GFR) were combined with four nephron number scenarios (no loss [2 M for Healthy, 1.33 M for Hyperfiltering, and 0.67 M for CKD kidneys], one-third, one-half, and two-thirds loss) to produce 16 scenarios. From each of these the maximum fold increase in biomarker concentration was extracted. The iso-intensity lines of fold increase were then interpolated. All scenarios are for a peak at 6 hours (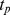), half the total excretion occurs within 12 hours (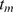), and a scaling factor (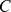) of 1000.

As with pre-formed biomarkers, the concentration of an induced biomarker depends on the number of nephrons assuming the same rate of induction of biomarker per nephron for each scenario. Hence, the Non-diabetic Hyperfiltering scenario is the same as the Healthy scenario for the same number of functioning nephrons (Figure 7). The CKD scenarios produced greater concentrations despite fewer nephrons contributing less total biomarker mass. This is because reduced urine output resulting from lower GFR increased biomarker concentrations substantially (see equation 21).

#### Filtered

**Figure 8:**
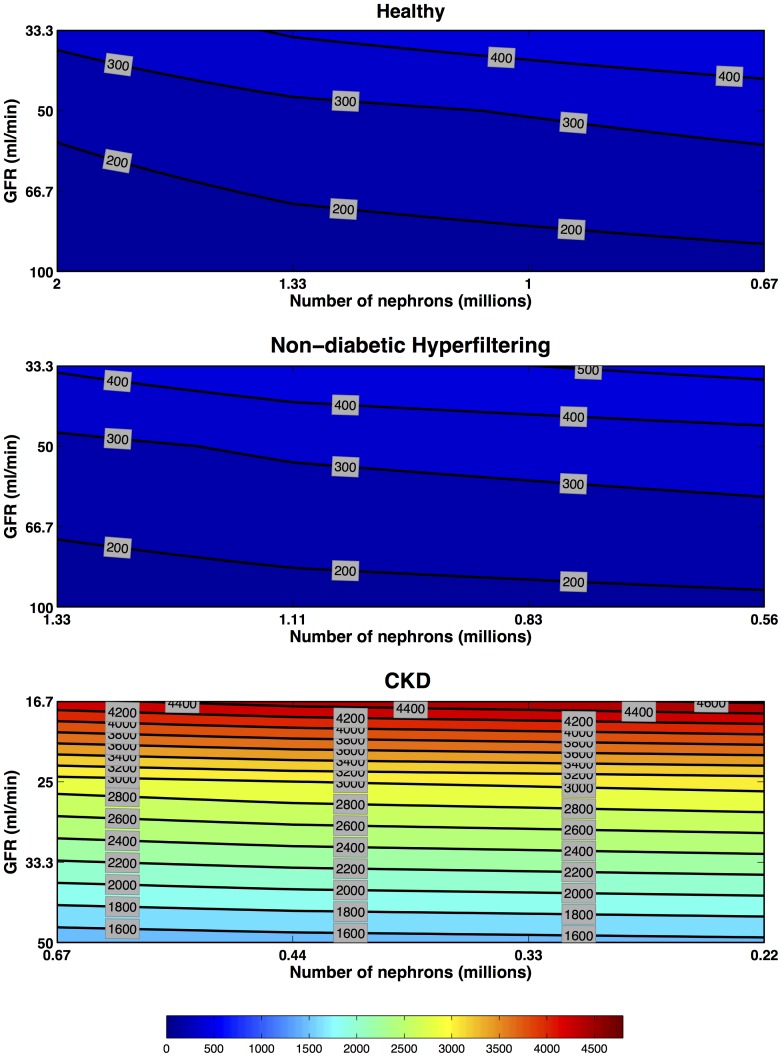
Filtered Biomarkers: multiple-nephron maximum fold concentration increase. Filtered biomarker concentration increases in Healthy, Hyperfiltering and CKD kidneys. Each line of iso-intensity represents the maximal fold increase of a preformed biomarker relative to the pre-injury concentration in a Healthy Kidney with 2 million nephrons and a GFR of 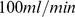. Four GFR scenarios (no change in GFR [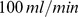 for Healthy and Hyperfiltering kidneys, and 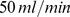 for CKD kidney], one-third, one-half, and two-thirds reduction in GFR) were combined with four nephron number scenarios (no loss [2 M for Healthy, 1.33 M for Hyperfiltering, and 0.67 M for CKD kidneys], one-third, one-half, and two-thirds loss) to produce 16 scenarios. From each of these the maximum fold increase in biomarker concentration was extracted. The iso-intensity lines of fold increase were then interpolated. All scenarios are for a receptor production rate constant 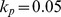 and loss rate constant 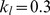.

**Figure 9:**
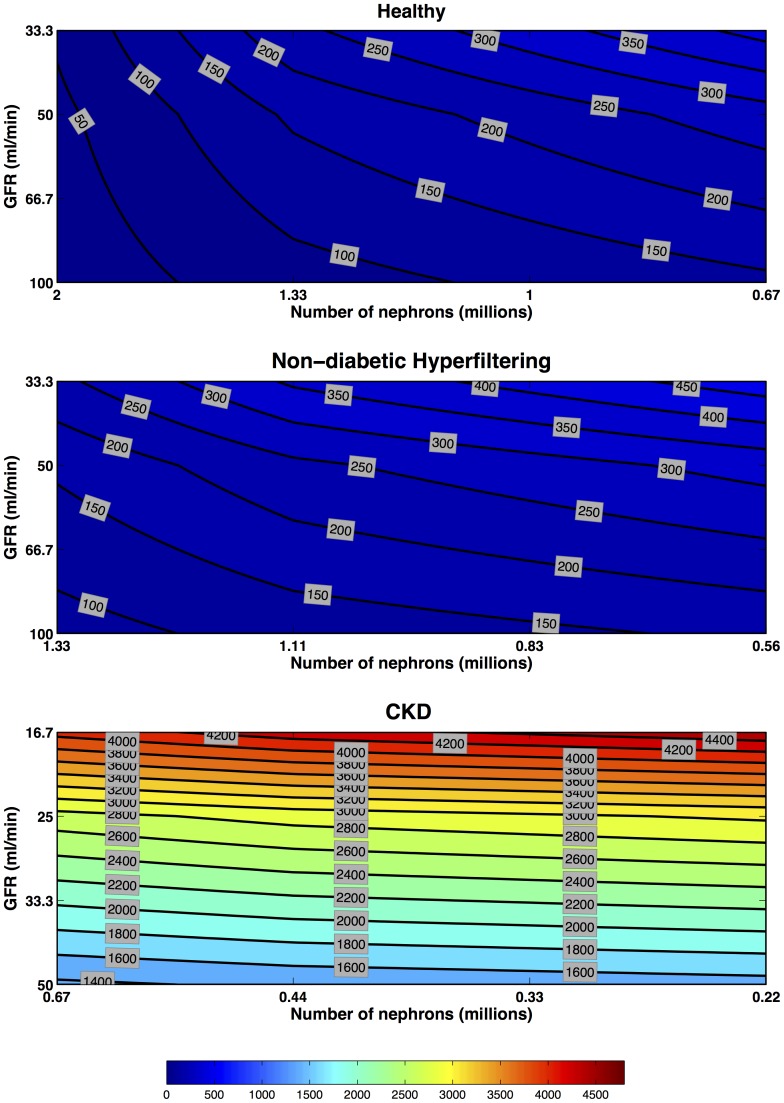
Filtered multiple-nephron fold concentration increase at 48 hours post-insult. Filtered biomarker concentration increases in Healthy, Hyperfiltering and CKD kidneys. Each line of iso-intensity represents the fold increase 48-hours following insult of a preformed biomarker relative to the pre-injury concentration in a Healthy Kidney with 2 million nephrons and a GFR of 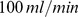. Four GFR scenarios (no change in GFR [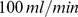 for Healthy and Hyperfiltering kidneys, and 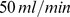 for CKD kidney], one-third, one-half, and two-thirds reduction in GFR) were combined with four nephron number scenarios (no loss [2 M for Healthy, 1.33 M for Hyperfiltering, and 0.67 M for CKD kidneys], one-third, one-half, and two-thirds loss) to produce 16 scenarios. From each of these the fold increase in biomarker concentration at 48-hours following insult was extracted. The iso-intensity lines of fold increase were then interpolated. Each scenario from figure 2 is represented for no change in GFR, one-third, one-half, and two-thirds reduction. All scenarios are for a receptor production rate constant 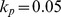 and loss rate constant 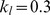.

Unlike pre-formed or induced biomarkers, the concentrations of filtered biomarkers which are normally reabsorbed within the tubules **increase** with decreasing nephron number (Figure 8). In addition, urinary concentrations also depend on plasma concentrations for filtered biomarkers. Because GFR is assumed to fall at time zero and remain low in these scenarios the plasma concentrations increase during the time interval shown. Consequently only in the Healthy Kidney when there is no change in nephron number, but merely a temporary reduction in reabsorption, does the urinary concentration return towards normal levels after 48 hours (Figure 9). In the CKD scenario there was only a small reduction below maximum concentrations by 48 hours.

#### NGAL

**Figure 10:**
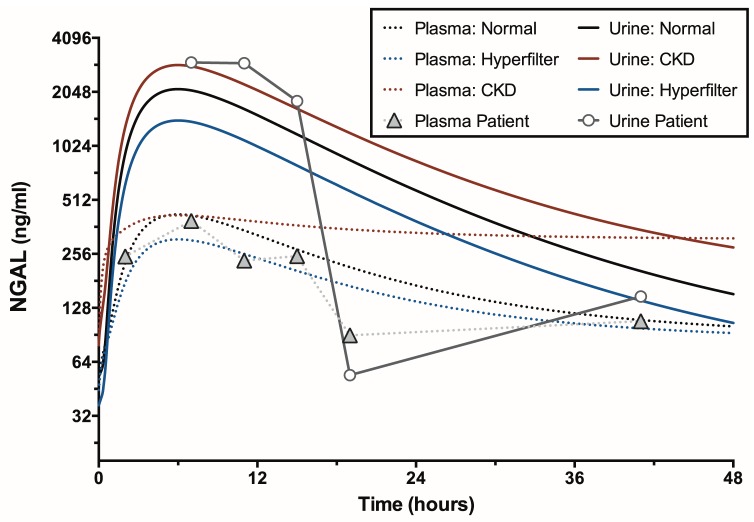
NGAL time-courses. Urinary and plasma NGAL concentration time-courses for the scenario of a 50% loss of GFR with no additional loss of nephrons for the Healthy (black lines), Hyperfiltering (blue lines) and CKD kidneys (red lines). Measured plasma and urinary NGAL values following a cardiac arrest in a 90 kg male with a history of hypertrophic obstructive cardiomyopathy and severely impaired left ventricular function who suffered a cardiac arrest in the emergency department and subsequently lost approximately 70% of GFR (Case A in [14]).

Figure 10 shows the time-courses of urinary and plasma NGAL concentrations for the Healthy, Non-diabetic hyperfiltering, and the CKD scenario after loss of two thirds of GFR without further loss of nephrons. Also shown are the measured plasma and urinary NGAL concentrations in a 90 kg male following cardiac arrest where creatinine changes indicated approximately 70% GFR reduction (more details are given as Case A in [14]). As with plasma creatinine kinetics, a two thirds loss of GFR is expected to result in a three-fold elevation in plasma NGAL [15], [16]. However, the typical increase in plasma NGAL exceeded this threshold suggesting that an increased rate of release of NGAL into the plasma. In these models, we set the rate of NGAL release into the plasma at 50 times that released into the tubules. This resulted in a 10 fold maximal increase in plasma NGAL concentration. The fold increase in urinary NGAL concentration was much greater (over 100 fold) [17].

## Discussion

This modeling exercise revealed potentially important differences in the time-course profiles of pre-formed, induced, and filtered urinary biomarkers of AKI which would be difficult to identify empirically and which, if validated experimentally and in human subjects, have clinical consequences. Pre-formed biomarkers respond quickest to injury, but have only very brief periods of elevation even when injury is ongoing. The shape of the induced biomarker time-course is influenced by the specific physiology associated with each induced biomarker while the fold-increase in concentration depends on a balance between the severity of the insult, which increases single nephron excretion, the number of remaining nephrons, to which total excretion is in proportion, and the extent of GFR reduction, which increases total biomarker concentration because of water reabsorption. Filtered biomarkers have the longest time-courses because of the increased plasma levels following GFR decrease. Somewhat surprisingly, peak concentrations are less dependent on the number of remaining nephrons than with induced biomarkers, and there is a small increase in peak-concentration with fewer nephrons. The peak concentration and profile depend on the degree of damage to the reabsorption mechanism and rate of recovery. Rapid recovery may be detectable through a rapid reduction in urinary concentration of a filtered biomarker that is normally reabsorbed. We also demonstrated that for all biomarkers, an inability to hyperfilter substantially increases peak biomarker concentrations, especially where there is substantial loss of nephron number due to chronic illness.

A single nephron will release pre-formed biomarkers into the lumen as long as injury continues and biomarker mass remains. The duration of insult is likely a combination of the duration of hypoxia and duration of reperfusion and associated inflammation. As hypoxia is a common cause of brush border damage, we speculate this may contribute to the early release of these pre-formed biomarkers [18], [19]. The duration of injury does not necessarily equate to the duration of a decreased GFR. Total biomarker excretion is limited by the pre-formed biomarker mass and the rate of regeneration. With a large available biomarker mass a very rapid increase in urinary concentration will follow injury. More severe injury will increase the rate of excretion and the peak excretion rate, but also reduce the duration over which excretion occurs (Figure 3). Urinary concentration increases with increasing nephron number because of the greater total available biomarker mass, and also increases with reduced GFR because of increased water reabsorption (Figure 6). The pre-formed biomarker concentrations for the Hyperfiltering and Healthy kidney models' were similar because hyperfiltration compensated for the loss of nephrons. However, in CKD kidneys, the increased water reabsorption increased biomarker concentrations. The time-courses of urinary biomarker concentrations demonstrates that the most rapid increases in AKI are shown by pre-formed biomarkers 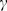-GGT, ALP, 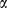-GST and 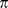-GST [2], [20]. Shortly following insult, high concentrations are diagnostic of later increases in plasma creatinine [2]. The “window of detectability” during which pre-formed biomarker excretion can be used to diagnose AKI is short. If the total available biomarker mass is sufficiently reduced, and the rate of regeneration is low, total excretion may fall to levels below that in an uninjured kidney (Figure 3). This may explain why 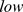 ALP concentrations in the EARLYARF trial at 36 hours post insult were diagnostic of AKI [2].

For induced biomarkers the single nephron excretion may continue indefinitely; mathematically, there is no upper limit to the biomarker mass excreted. In practice, the total mass excreted depends on physiological factors that are not well understood. However, total excretion probably depends on the severity of the insult. We have shown elsewhere that total excretion of several biomarkers is related to AKI severity stage and need for dialysis and death [21]. Nevertheless, the reported time-courses [2], [3], [22] suggest, that total biomarker and peak biomarker excretion are limited in duration and follow an approximately log-normal curve over time. This also appears true of gene-expression of upregulated biomarkers like NGAL [23]. The peak concentrations in the multiple nephron scenarios, as with pre-formed biomarkers, increase with the available nephrons, increase with lower GFR, and are greatest in CKD patients. Induced biomarkers have differing time-courses, with some reaching a peak much later than others, for example KIM-1 increases more slowly than NGAL or IL18 [24]. This may be modelled by varying the time to maximal biomarker concentrations in the equations for a log-normal distribution of gene expression. In figure 4 the dotted curve illustrates a biomarker with later peak excretion compared to other biomarkers. Over the first few hours the change in concentration is minimal. This mimics the physiological response of KIM-1, which is involved in the phagocytosis of dead cells in the post-ischemic kidney [25].

The urine concentrations of filtered biomarkers depend on both changes in reabsorption rate, which may be low (e.g. for creatinine), or very high (e.g. for cystatin C), and on the plasma concentration. The temporal profile for a filtered and normally reabsorbed urinary biomarker, like cystatin C, is likely to increase rapidly because of damage to the megalin-cubulin receptors and/or competition for reabsorption [26]. The plasma concentration of such a biomarker increases with time while GFR is reduced which in turn maintains an elevated urinary concentration [2], [27]. Unlike pre-formed and induced biomarkers, filtered biomarker concentrations increase with decreasing nephron number because less biomarker mass is reabsorbed. If recovery of GFR is accompanied by recovery of reabsorption then urinary concentrations of a filtered and normally reabsorbed biomarker will reduce more rapidly than their plasma concentrations or plasma creatinine. Thus a filtered and normally reabsorbed urinary marker should be an earlier marker of recovery than a plasma biomarker.

Data in healthy populations of pre-insult normal biomarker concentrations of most of the candidate structural injury biomarkers is sparse. Pennemen and colleagues measured urinary concentrations of KIM-1, NAG, NGAL, and cystatin C in 338 non-smoking healthy volunteers between the ages of 0 and 95 [12]. They noted some sex and age related differences in mean concentrations, but these were diminished when values were normalised to urinary creatinine. Cullen and colleagues measured NGAL in 174 adults and noted age related differences [28]. Other studies have control subjects which provide a pseudo-normal range, for example for IL-18 [29]. This lack of healthy population data needs to be addressed, if only to establish reference ranges. In this study we have deliberated avoided presenting biomarker concentrations, except for the NGAL case study. Instead we presented fold increases from which concentrations may be calculated if a pre-insult concentration is known.

Urinary NGAL is both induced and filtered; it is released into the distal tubule following insult and simultaneously enters the circulation increasing plasma NGAL (which may also increase with systemic bacterial infection) from where it is also filtered where it may be reabsorbed by the megalin-cubulin receptors [17], [30], [31]. That portion of filtered NGAL not reabsorbed in the proximal tubule (which may be damaged) will reach the final urine. Thus circulating NGAL may increase the duration of increase in the urine beyond that of direct tubular release. We have shown recently that plasma NGAL performs partly as a biomarker of function and partly as a biomarker of structural injury [32]. The plasma concentration typically increases to more than can be explained simply by a loss of GFR and subsequent increase in filtered analyte concentrations. As NGAL is distributed over at least the plasma volume this requires an increase in NGAL production, which may be systemic, from other organs, or from the kidney itself. Our simulation suggests the proportion of induced NGAL released into the circulation must be many times that released into the tubules. This observation begs the question concerning where the NGAL appearing in the plasma is produced and how it enters the plasma? If it is primarily produced in the kidney, then we can be more confident that plasma NGAL relates to kidney injury rather than injury to other organs or a systemic source. One consequence of the kinetics is that whilst urinary NGAL and plasma NGAL may peak at approximately the same time, urinary NGAL concentrations will return to normal more rapidly.

This analysis is subject to the limitations imposed by the model assumptions. For pre-formed biomarkers we assumed that the rate of loss (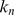) was constant for the whole period of the insult. This may be true for well defined insults such as a cardiac arrest or surgery. With other causes of AKI, for example sepsis, this is likely to vary, which may result in a later peak excretion rate and a broadening of the temporal profile. In all the multi-nephron scenarios we could not account for the ‘dead’ space in the renal pelvis and ureters. From a practical perspective the initial increases in biomarker concentration will be delayed by the time necessary for biomarker to reach the bladder. This will also broaden the temporal profile since not all nephrons are of equal length. We modelled hyperfiltration only for the non-diabetic case where there is loss of nephrons. In diabetic kidney disease, hyperfiltration without loss of nephron number occurs in the early stages of the renal involvement. When GFR is elevated above that of the healthy kidney, the filtered and induced biomarker profiles will only differ from the healthy kidney if the urine flow rate is elevated. In this case, the biomarker concentrations will be lower in proportion to increased urine flow. While the filtered biomarker profiles will be similarly affected by urine flow rate, increased filtration may increase the total filtered biomarker excretion rate. In all scenarios concentration varies with retention of water. This may be artificially varied in the clinic either through the introduction of a fluid bolus or through loop-diuretics. In both cases the urine is likely to become more diluted and the fold increase reduced. Normalising biomarkers to urinary creatinine has been proposed and discussed in the literature as a way to account for variations in water retention [21], [33]. However, we note that urinary creatinine itself is a filtered biomarker with an element of secretion into the tubules, so will be affected by GFR and nephron number differently from other urinary biomarkers. Effectively, this adds noise to a biomarker normalised to creatinine signal meaning that the ratio threshold for diagnosis would need to be greater.

We modelled nephron number as decreased immediately after insult. It seems more likely, that loss of filtration may be slowed progressively, but there is no data to confirm this. The likely effect is broadening of the temporal profiles presented. We also assumed that the water reabsorption rate was constant during AKI and modelled only a step decrease in GFR. As Moran and Myers demonstrated with creatinine kinetics [15], the temporal profiles are likely to change as GFR changes. A change in profile will be greatest for filtered biomarkers.

The ultimate utility of each type of biomarker in each clinical scenario will depend on our understanding of the time-course profiles. Our modeling exercise has highlighted that these will depend on pre-insult GFR, nephron number and renal reserve. As with all modeling exercises, we are limited by the assumptions. What is needed are experimental and clinical studies which measure time-course profiles of multiple biomarkers under known scenarios of GFR, nephron number and renal reserve. Until then, therefore, our conclusions remain speculative. We predict that preformed biomarkers are the earliest indicators of kidney injury, but their brief and early windows of detectability are easily missed in clinical scenarios. Peak urinary concentrations reflect severity of insult, however the temporal profile is reduced by more severe insults. Induced biomarkers have varying durations and times to peak concentration. The window of detectability is extended by the duration and severity of injury. Filtered biomarkers reflect both injury and change of function and have a the broadest time-courses. In a clinical scenario, biomarker choice depends on when measurement is made in relation to the timing of the renal insult, and biomarker interpretation depends on an understanding of the pre-insult kidney size (nephron number) and function (hyperfiltering or not).
